# Utility of the Shock Index as a Prognostic Predictor in Patients Undergoing Emergency Surgery for Trauma: A Single Center, Retrospective Study

**DOI:** 10.3390/jcm14196783

**Published:** 2025-09-25

**Authors:** Byungchul Yu, Chun Gon Park, Kunhee Lee, Youn Yi Jo

**Affiliations:** 1 Department of Trauma Surgery, Gil Medical Center, Gachon University College of Medicine, Incheon 21565, Republic of Korea; 2 Department of Anesthesiology and Pain Medicine, Gil Medical Center, Gachon University College of Medicine, Incheon 21565, Republic of Korea

**Keywords:** shock index, trauma, emergency surgery, mortality

## Abstract

**Background:** Shock index (SI) is calculated by dividing heart rate (HR) by systolic blood pressure (sBP) and is a useful tool for predicting the prognosis of trauma patients. This study aimed to determine whether SI is useful in predicting mortality in patients undergoing emergency surgery for trauma. **Methods**: We analyzed 1657 patients who underwent emergency surgery for trauma. Patients were divided into SI < 1 and SI ≥ 1 groups and the Glasgow Coma Scale (GCS), Injury Severity Score (ISS), revised trauma score (RTS), Korean Triage and Acuity Scale (KTAS), transfusion amount, and mortality were compared. Binary logistic regression analysis was performed to identify factors associated with mortality. **Results**: There were significant differences in GCS, ISS, RTS, and KTAS in the SI ≥ 1 group compared to the SI < 1 group (all *p*-values < 0.001). In the SI < 1 cohort, the mortality rate was 11% (144/1283), and in the SI ≥ 1 group the mortality rate was 33% (125/374) (*p* < 0.001). Age, GCS, ISS, SI ≥ 1, and KTAS were determined to be predictors of mortality by logistic regression analysis. In particular, SI ≥ 1 group members exhibited a high association with elevated mortality (OR, 2.498; 95% CI, 1.708–3.652; *p* < 0.01). **Conclusions**: Although SI alone has limitations in predicting the patient’s prognosis, patients with SI ≥ 1 upon arrival at the emergency room are associated with mortality of patients undergoing emergency surgery for trauma, along with already known trauma assessment systems such as GCS, ISS, and KTAS.

## 1. Introduction

In acute injuries such as trauma, it is very important to immediately assess the severity of the patient’s damage and establish a treatment plan in the early stages. As it takes time and cost to conduct blood or imaging tests, various tools for the immediate judgment of injury severity have been studied. Rapid surgical decision-making and implementation through the acute care surgery system can improve the prognosis of trauma patients through rapid hemostasis, prevention of injury expansion, and repair of injury [1].

The shock index (SI) is calculated by dividing the heart rate (HR) by the systolic blood pressure (sBP) and can rapidly determine the patient’s condition [2]. While sBP or HR alone can distort the patient’s condition due to increased catecholamine levels or compensatory mechanisms in stressful conditions [3]. HR increases and sBP decreases during acute hemorrhage, but fluctuates erratically across various stimuli and, because baseline values before hemorrhage is often unknown in clinical settings, absolute HR and sBP alone has limited utility in predicting the extent of injury [3]. SI is known to reflect the patient’s condition relatively accurately even in situations where HR and BP are normal because it is related to ventricular function and circulatory volume [2,4].

A previous meta-analysis involving 348,687 patients with trauma demonstrated that SI ≥ 1.0 at hospital arrival is associated with higher in-hospital mortality (pooled risk ratio 4.15; 95% CI 2.96–5.83) than SI < 1 [4]. In addition, an analysis of 217,190 participants (age ≥ 65 years) reported that SI ≥ 1.0 is associated with higher requirements for blood product transfusions (*p* = 0.001) and greater in-hospital morbidity (*p* = 0.02) than SI < 1 [5]. Although, blood pressure is lower and heart rate is faster in preschool-age children, than in adults, so the normal value of SI is bound to be different and, tachycardia appears early in acute hemorrhage, while delayed hypotension appears, so age-adjusted SI is used, unlike in adults [6], elevated (age adjusted) SI is significantly associated with intensive care unit (ICU) admission, requirement for mechanical ventilation, blood transfusions, and in-hospital mortality [7].

As mentioned above, the SI is a useful tool for predicting the prognosis of patients with trauma. However, no previous studies have investigated whether SI is helpful in predicting the prognosis of patients who undergo immediate emergency surgery for trauma. Therefore, we analyzed whether SI was helpful in predicting the prognosis of patients who underwent emergency surgery for trauma.

## 2. Materials and Methods

### 2.1. Ethical Considerations and Participants

This retrospective study was approved by the Institutional Review Board (IRB) of Gachon University Gil Hospital. Among 1666 patients who were admitted directly from the emergency room to the operating room for emergency surgery due to trauma at our institution, a level I trauma center, 1657 patients were included in the analysis, excluding 9 patients whose cause of trauma was slipping or crushing injury due to acute cerebrovascular attack, ruptured cerebral aneurysm, or sudden worsening of underlying disease, from January 2019 to December 2024.

All included patients were immediately transported to the emergency room (ER) from the site of trauma, and resuscitations including fluid and/or blood component infusion were performed after securing an intravenous line upon arrival at the ER. Patients who were transferred from a local hospital after resuscitation were excluded. Damage caused by vulnerability due to worsening of underlying disease, acute cerebral infarction or cerebral hemorrhage (non-traumatic) were excluded. Whether immediate surgical treatment was required was determined by a trauma specialist in the emergency room. Postoperative ICU admission was determined by the judgment of trauma surgeons and anesthesiologists. Priority for ICU admission was given to patients requiring mechanical ventilator care after surgery, hemodynamic instability, and the need for additional surgery or intervention after damage control surgery. Although preoperative evaluation for underlying medical conditions and preparation of patients as for elective surgery was not performed, all patients received ongoing treatment for underlying diseases, including treatment of traumatic injuries, and additional testing and support as appropriate after surgery.

### 2.2. Analyzing Variables

Immediately after arrival at the ER, measurable indicators, such as age, sex, sBP, HR, respiratory rate (RR), and cause and extent of trauma, were recorded. The time from the patient’s arrival at the ER to admission to the operating room (ER duration) was measured. This refers to the time required for physical examination, imaging studies, laboratory tests, to determine the patient’s condition and injury and for initial resuscitation. Patients’ state of consciousness was assessed using the Glasgow Coma Scale (GCS), and patients were classified using the Korean Triage and Acuity Scale (KTAS; level I, Resuscitation; level 2, emergent; level 3, urgent; level 4, less urgent; level 5, not urgent) [8]. Based on the above variables, the injury severity score (ISS), revised trauma score (RTS, RTS = 0.9368 × GCS coded points + 0.7326 × SBP coded points + 0.2908 × RR coded points) [9], and SI (HR/sBP) were calculated.

The amount of transfused blood within the initial 4 h after the patient’s arrival at the emergency room and the additional amount of transfused blood over the next 24 h were recorded. In addition, we recorded whether the patient was admitted to the ICU or general ward after surgery and whether the patient died during hospitalization (in-hospital mortality).

The primary endpoint of this retrospective analysis was the relationship between SI and in-hospital mortality among patients who underwent emergency surgery for trauma.

### 2.3. Statistical Analysis

Data were analyzed using the SPSS v.22 software (SPSS Inc., Chicago, IL, USA). To compare clinical data between groups with SI < 1 and those with SI ≥ 1, independent Student’s *t*- or Mann–Whitney *U*-tests were performed for continuous variables, and Chi squared or Fisher’s exact test were performed for categorical data. Results are described as means ± standard deviation, number of patients (%), or median [interquartile range]. Binary logistic regression analysis was performed to identify factors associated with mortality. The factors applied in the logistic regression analysis were age, GCS, ISS, RTS, and KTAS scores. As preoperative vital signs were already included in the calculation formulae for the preceding variables, they were excluded from analysis. In addition, we separately classified patients with severe trauma (ISS > 15) and performed logistic regression analysis. Statistical significance was set at *p*-values < 0.05.

## 3. Results

The patient demographics and ER data are presented in Table 1. Among a total of 1657 patients, 374 (23%) had SI ≥ 1. There were statistically significant differences in age, cause of trauma, sBP, HR, and RR between the two groups. The time spent in the emergency room before surgery was statistically significantly shorter in the SI ≥ 1 group than in the SI < 1 group (*p* < 0.001).

The severity indices of patients evaluated in the ER are presented in Table 2. There were significant differences in GCS, ISS, RTS, and KTAS in the SI ≥ 1 group compared to the SI < 1 group (all *p*-values < 0.001).

The amounts of blood transfused, ICU admission, and mortality are presented in Table 3. A total of 78% (1289/1657) of patients (72% in the SI < 1 group and 98% in the SI ≥ 1, *p* < 0.001) were admitted to the ICU after surgery. The overall in-hospital mortality rate was 16% (269/1657). In the SI < 1 group, the mortality rate was 11% (144/1283), and in the SI ≥ 1 group, it was 33% (125/374) (*p* < 0.001).

Logistic regression analysis to identify factors associated with mortality is presented in Table 4. All factors had *p* values of <0.01 by univariate regression analysis, and, in multivariate regression analysis age, GCS, ISS, SI ≥ 1, and KTAS were determined to be predictors of mortality. In particular, SI ≥ 1 exhibited a high association with elevated mortality (OR, 2.498; 95% CI, 1.708–3.652; *p* < 0.01). The variance inflation factors of each variables were 1.024 for age, 2.137 for GCS, 1.525 for ISS, 1.253 for RTS, 1.202 for SI, and 2.016 for KTAS. Nagelkerke R square and value of Hosmer and Lemeshow test of regression analysis were 0.448 and 0.202, respectively.

Among all patients, 965 had an ISS > 15 and 255 of them died, accounting for 95% (255/269) of all deaths. In subgroup analysis of the factors in Table 5 for patients with an ISS > 15, all factors in the univariate regression analysis were *p* < 0.05. In multivariate regression analysis, age, GCS, ISS, SI ≥ 1, and KTAS were determined to be predictors of mortality and were found to be statistically significant. Even in patients with severe trauma with ISS > 15, SI ≥ 1 was found to be closely associated with mortality (OR, 2.706; 95% CI, 1.845–3.968; *p* < 0.001).

## 4. Discussion

In this study, which involved only patients who underwent emergency surgery for trauma, SI was found to be a good predictor of patient mortality, even in patients with severe injury and an ISS > 15. Although previous studies have analyzed the association between SI and patient prognosis, to our knowledge, our study is the first to analyze only patients who underwent emergency surgery for trauma.

As SI is calculated only from sBP and HR, it is a simple and useful tool that can rapidly determine a patient’s condition within seconds without additional tests or interpretations. The SI was first proposed by Allgöwer et al. in 1967 [10] as a tool that was calculated based on the mechanism of a decrease in BP and an increase in HR as a compensatory mechanism when the circulating blood volume suddenly decreases in acute hemorrhagic shock. In a recent echocardiographic study of 6289 cardiac ICU patients (58% with acute coronary syndrome), increased SI was closely associated with decreased left ventricular (LV) systolic function, such as LV ejection fraction (EF) [11]. Increased SI is also associated with worsening right ventricular (RV) function indices [11].

The normal value of SI is 0.5–0.7, and 1.0 or higher is applied to predict morbidity or mortality and apply massive transfusion protocols [12]. A recent study analyzing 5958 trauma patients showed that SI > 0.7 was helpful in predicting the need for transfusion, and a cutoff value of SI = 1 resulted in the best positive and negative predictive values for predicting compensatory shock in normotensive trauma patients [13]. In a meta-analysis [4] of adult trauma patients, all studies showed that SI ≥ 1 has a significantly higher in-hospital mortality rate compared to SI < 1. In our study, the amount of transfusion within the first 4 h after ER presentation and up to 24 h thereafter was significantly higher in the group with SI ≥ 1, and mortality was also higher (11% vs. 33%, *p* < 0.001) in the group with SI ≥ 1 than in SI < 1.

We analyzed the utility of the SI only in patients who underwent emergency surgery for trauma. However, the SI itself can help promptly determine the need for surgery [14]. In an analysis of 4008 military trauma patients in a modern battlefield, the need for emergency surgical procedures was statistically significantly higher in the group with SI ≥ 0.8 than in the group with SI < 0.8 (30.7% vs. 6.5%, *p* < 0.001) [14]. Furthermore, regression analysis confirmed that SI ≥ 0.8 was an independent risk factor for the need for emergency surgery [14]. In an analysis of 1645 patients with blunt chest trauma, high SI cases required more chest interventions and were associated with more blood transfusions [15]. In addition, a high SI was an independent predictor of mortality (OR = 3.506; 95% CI = 1.389–8.848; *p* = 0.008), which was consistent after adjustment for chest intervention (OR 2.923; 95% CI 1.146–7.455; *p* = 0.02) [15].

There are many scoring systems for assessing the severity of trauma which are useful in predicting a patient’s prognosis or determining treatment directions [16,17,18,19]. In a study involving 32,201 trauma patients who required emergency interventions, such as massive transfusion, resuscitative procedures, or surgery, the combination of SI and GCS was an effective predictor of emergency interventions [16]. In another multicenter study including 318,506 trauma patients, a reverse SI multiplied by the GCS (rSIG) cutoff values of 16.5 was determined as a predictor of in-hospital mortality, and the authors proposed that a high rSIG value is associated with in-hospital mortality in patients with traumatic brain injury [17]. Various tools, such as the ISS, RTS, and GCS, have been proven to evaluate patient severity and predict prognosis in large cohort studies and research results have shown that using these tools together with the SI helps predict patient prognosis [18,19]. In a study comparing ISS, trauma index, SI, modified SI, and age-adjusted SI to predict early mortality in trauma patients, SI showed the greatest reference value for predicting mortality in patients with traumatic shock. In addition, SI, along with MSI and ASI, was found to be good indicator for evaluation index when measuring blood loss after trauma [18]. Our study also showed lower GCS, RTS, and higher ISS in the group with SI ≥ 1, which is consistent with previous studies. In a study which evaluated the validity of the KTAS for predicting 30-day mortality in severe trauma (ISS ≥ 16) patients, lower KTAS was associated with higher 30-day mortality [20]. Although the KTAS is a useful tool for predicting the mortality rate of patients with trauma, it has not been studied in conjunction with the SI. Therefore, differences according to the SI groups presented in the results of this study might have clinical implications. In a logistic regression analysis to identify factors related to mortality, GCS, ISS, SI, and KTAS, excluding RTS, were statistically significant, and the same results were obtained when only patients with severe trauma with ISS >15 were included. It can be predicted that the reason for this contradictory result is that the RR value used in RTS calculation is statistically significantly higher in the SI ≥ 1 group.

In the results of our study, GCS, sBP, and RR, which are necessary for calculating RTS, all showed statistically significant differences between the SI ≥ 1 group and SI< 1 group, and although they were also significant in univariable regression analysis, they failed to produce meaningful results in multivariable regression analysis. Previous studies had shown that RTS was a good predictor of mortality in trauma patients [9,19], but unlike our study, there were no studies limited to patients who underwent emergency surgery, so further research might be necessary.

It is known that patients with a normal SI upon arrival at the ER but a high prehospital shock index have a higher 24 h mortality rate [21]. Our study only applied SI upon arrival at the ER and only included patients who underwent emergency surgery, so it did not reflect changes in prehospital SI or SI after resuscitation. However, repeated measurements of SI and changes in trends can be useful in predicting patients’ responses to treatment [22].

This retrospective study had certain limitations. First, an SI below the threshold value does not guarantee the absence of mortality or morbidity. As SI < threshold alone has low sensitivity, it should be used to predict prognosis together with other indicators, and even if SI is low, it should not be used to exclude risk [23]. Second, the predictive strength of the SI may be diminished in patients with cardiovascular disease due to compromised normal compensation mechanisms of blood pressure or HR [24]. In patients with uncontrolled hypertension, an increase in baseline blood pressure or the use of antihypertensive agents such as beta-blockers or calcium channel blockers can attenuate changes in blood pressure and HR in hypovolemic states [25]. Although underlying medical conditions such as cirrhosis, congenital coagulopathy, ischemic heart disease, chronic obstructive pulmonary disease, and diabetes can affect the mortality of trauma patients [26], the exclusion of preoperative medical conditions from our analysis is also a limitation of this study because they were often not properly identified in emergency surgical settings.

## 5. Conclusions

Although SI alone has limitations in predicting the patient’s prognosis, patients with SI ≥ 1 upon arrival at the emergency room are associated with in-hospital mortality of patients undergoing emergency surgery for trauma, along with already known trauma assessment systems such as GCS, ISS, and KTAS.

## Figures and Tables

**Table 1:** Patients’ demographic and emergency room data.

	Overall *n* = 1657	SI < 1 *n* = 1283	SI ≥ 1 *n* = 374	*p*-Value
Age, years	53 ± 19	54 ± 19	50 ± 20	<0.001
Sex, M/F	1234/423	960/323	274/100	0.292
Trauma causes				<0.001
Traffic accidents	622 (38)	461 (36)	161 (43)	
Stab injury	235 (14)	189 (15)	46 (12)	
Crush	133 (8)	115 (9)	18 (5)	
Slip down	230 (14)	216 (17)	14 (4)	
Fall down	313 (19)	194 (15)	119 (32)	
Machine	55 (3)	44 (3)	11 (3)	
Others	26 (2)	25 (2)	1 (0)	
unknown	43 (3)	39 (3)	4 (1)	
sBP, mmHg	129 ± 42	144 ± 32	79 ± 33	<0.001
HR, beats/min	93 ± 26	87 ± 19	114 ± 34	<0.001
RR, breaths/min	21 ± 5	21 ± 4	23 ± 8	<0.001
ER duration, min	131 ± 110	143 ± 120	93 ± 54	<0.001

Values are presented as mean ± SD, the number of patients (%). sBP, HR, RR, systolic blood pressure, HR, and respiratory rate upon arrival at the emergency room; ER duration, length of stay in the emergency room.

**Table 2:** Indices for patient severity assessment in the emergency room.

	Overall *n* = 1657	SI < 1 *n* = 1283	SI ≥ 1 *n* = 374	*p*-Value
Glasgow coma scale	15 [12–15]	15 [14–15]	14 [7–15]	<0.001
Injury severity score	17 [9–26]	16 [9–25]	27 [17–34]	<0.001
Revised trauma score	8 [7–8]	8 [8–8]	7 [6–10]	<0.001
KTAS	2 [2–3]	2 [2–3]	2 [1–2]	<0.001

Values are presented as median [interquartile range]. KTAS, Korean Triage and Acuity Scale.

**Table 3:** Perioperative data.

	Overall *n* = 1657	SI < 1 *n* = 1283	SI ≥ 1 *n* = 374	*p*-Value
pRBC 4 h, pint	2.5 ± 4.4	1.3 ± 2.6	6.6 ± 6.4	<0.001
pRBC 24 h, pint	1.3 ± 3.0	1.0 ± 2.0	2.6 ± 4.8	<0.001
Postop ICU admission, n	1289 (78)	924 (72)	365 (98)	<0.001
Mortality	269 (16)	144 (11)	125 (33)	<0.001

Values are presented as means ± SD or number of patients (%). pRBC at 4 h and 24 h, transfusion of packed red blood cells during the initial 4 h and additional transfusion at 4–24 h after emergency room admission.

**Table 4:** Logistic regression analysis to identify factors associated with mortality.

	Univariable			Multivariable		
	OR	95% CI	*p*-Value	OR	95% CI	*p*-Value
Age	1.018	1.010–1.025	<0.001	1.033	1.022–1.044	<0.001
Glasgow coma scale	0.740	0.715–0.766	<0.001	0.834	0.792–0.879	<0.001
Injury severity score	1.089	1.076–1.103	<0.001	1.051	1.033–1.068	<0.001
Revised trauma score	0.775	0.709–0.847	<0.001	0.987	0.896–1.088	0.796
Shock index ≥ 1	3.971	3.013–5.233	<0.001	2.498	1.708–3.652	<0.001
KTAS	0.154	0.122–0.196	<0.001	0.518	0.363–0.739	<0.001

OR, odds ratio; 95% CI, 95% confidence interval; KTAS, Korean Triage and Acuity Scale.

**Table 5:** Risk factors associated with mortality in the severe injury group defined as injury severity score > 15.

	Univariable			Multivariable		
	OR	95% CI	*p*-Value	OR	95% CI	*p*-Value
Age	1.014	1.006–1.023	0.001	1.025	1.015–1.036	<0.001
Glasgow coma scale	0.788	0.760–0.818	<0.001	0.835	0.792–0.882	<0.001
Revised trauma score	0.857	0.790–0.930	<0.001	0.972	0.882–1.072	0.573
Shock index ≥ 1	2.262	1.684–3.039	<0.001	2.706	1.845–3.968	<0.001
KTAS	0.221	0.170–0.286	<0.001	0.516	0.355–0.750	0.001

OR, odds ratio; 95% CI, 95% confidence interval; KTAS, Korean Triage and Acuity Scale.

## Data Availability

Data is contained within the article.
